# Supplementary material for: Transcriptome Analysis Reveals Key Genes Involved in the Response of Pyrus betuleafolia to Drought and High-Temperature Stress
Source: Plants (Basel). 2024 Jan 20;13(2):309. doi: 10.3390/plants13020309 (PMC10819556; doi:10.3390/plants13020309)
Supplement: Supplementary file 1 [file plants-13-00309-s001.zip › plants-2772216-supplementary.pdf]

Supplementary Table S1 The HSPs which response to high-temperature stress in the RNA-seq data.

Gene ID	H_9h (FPKM)	H_0h (FPKM)	Annotation
LOC103927498	1338.02	0.09	heat shock protein-like
LOC103927502	10442.51	0.91	heat shock protein-like
LOC103927503	2748.583	0.046667	heat shock protein-like
LOC103927504	3953.337	0.21	heat shock protein-like
LOC103930915	51.42667	3.966667	heat shock protein 70 like
LOC103930933	46.66667	15.17333	heat shock protein 70
LOC103935290	1.7	0.486667	heat shock protein 70 like
LOC103935982	44.31667	5.096667	heat shock protein 70 8-like
LOC103936175	4038.977	4.18	18.1 kDa class I heat shock protein-like
LOC103936185	5237.453	10.26	18.1 kDa class I heat shock protein-like
LOC103936191	954.7133	3.016667	16.9 kDa class I heat shock protein 2-like
LOC103936199	496.8833	0.236667	18.1 kDa class I heat shock protein-like
LOC103936226	10476.14	19.18667	18.5 kDa class I heat shock protein-like
LOC103940225	340.3333	7.97	small heat shock protein, chloroplastic
LOC103942692	101.7467	11.15667	16.9 kDa class I heat shock protein 2-like
LOC103942700	9291.43	9.456667	16.9 kDa class I heat shock protein 2-like,
LOC103942709	1024.593	1.323333	16.9 kDa class I heat shock protein 2-like
LOC103943366	7.1	0	small heat shock protein, chloroplastic-like
LOC103945001	158.97	57.5	stromal 70 kDa heat shock-related protein
LOC103945002	263.5467	19	heat shock 70 kDa protein 6
LOC103945383	162.27	1.06	heat shock 70 kDa protein-like
LOC103945384	297.4	1.223333	heat shock 70 kDa protein-like
LOC103946362	255.1533	78.60667	stromal 70 kDa heat shock-related protein
LOC103946415	743.1867	0.08	16.9 kDa class I heat shock protein 1-like
LOC103946600	719.5733	167.1167	heat shock protein 90-5, chloroplastic
LOC103948411	436.3167	28.61667	heat shock cognate 70 kDa protein 2
LOC103948601	19.47	6.506667	17.4 kDa class III heat shock protein-like
LOC103948841	76.71	32.98333	heat shock 70 kDa protein 15-like
LOC103948842	65.77667	25.21667	heat shock 70 kDa protein 15-like
LOC103949050	47.88333	1.146667	heat shock 70 kDa protein-like
LOC103949874	499.0267	0.293333	small heat shock protein
LOC103951596	2278.11	22.08333	small heat shock protein
LOC103952262	188.5333	69.63333	heat shock protein 90-2
LOC103952522	2921.547	0.08	17.1 kDa class II heat shock protein-like
LOC103953301	115.9933	0.13	18.1 kDa class I heat shock protein-like
LOC103955091	114.8367	9.703333	heat shock protein 90-6, mitochondrial
LOC103955267	120.4833	0.303333	26.5 kDa heat shock protein, mitochondrial
LOC103956001	422.8233	14.55	heat shock 70 kDa protein
LOC103956116	92.09333	17.04333	heat shock 70 kDa protein, mitochondrial

LOC103956628	143.2633	1.57	heat shock 70 kDa protein 8-like
LOC103956927	145.2067	94	heat shock protein 90-2
LOC103957827	55.10667	20.58	heat shock 70 kDa protein, mitochondrial
LOC103958215	628.9533	7.32	heat shock protein 83
LOC103959434	516.5267	61.64	15.7 kDa heat shock protein
LOC103959693	244.7933	0	class I heat shock protein-like
LOC103959824	70.30333	0.783333	23.6 kDa heat shock protein
LOC103959948	0.57	0	15.7 kDa heat shock protein
LOC103960307	497.6167	0	22.0 kDa class IV heat shock protein
LOC103960954	333.4667	0	class I heat shock protein-like
LOC103961953	77.15	0.553333	23.6 kDa heat shock protein
LOC103963260	125.8533	4.67	17.6 kDa class I heat shock protein
LOC103964327	211.6767	0.21	heat shock protein 83-like
LOC103965881	146.2667	0	heat shock 70 kDa protein-like
LOC103966169	301.4367	0.026667	heat shock protein 83-like
LOC103966404	1.226667	6.83	heat shock 70 kDa protein 18-like
LOC108868142	23.53333	13.46	15.7 kDa heat shock protein

Supplementary Table S2 List of primers used in this study.

Primer	Sequence (5'-3')
LOC103947119	F: GTTCGGGGTTTCTGGATGC<br>R: TACTTGGGACGGTGGCAAG
LOC103950596	F: ACAAAAGATCCGCCAGCGT<br>R: ATCCGGACGCCGTATGATT
LOC103959917	F: TGTAGAGCAGTCAGCAATGTCCA<br>R: CTATTGACACCAGCAGCAGATGA
LOC103946681	F: AGCAGCGAAAGGTCTTCGA<br>R: TCTCACCAGCCGTTTGGAC
LOC103966009	F: AACCCCCATGAGATCAACAAGT<br>R: CAACGGTGGAGATATGACTTCG
LOC103960544	F: GCGAAACCGATAATGTCGC<br>R: GGAGCACGCGTAAGTCGAG
LOC103927985	F: TGTGGGAGCAAAGGATGGA<br>R: GCAAGCGTTTCTCAGCACC
PbrGAPDH	F: TGGTGTCATGGTTGGTATGG<br>R: CAGGAGCAACACGAAGTTCA
